# Group cohesion in foraging meerkats: follow the moving ‘vocal hot spot’

**DOI:** 10.1098/rsos.170004

**Published:** 2017-04-26

**Authors:** Gabriella E. C. Gall, Marta B. Manser

**Affiliations:** 1 Department of Evolutionary Biology and Environmental Studies, University of Zurich, Winterthurerstrasse 190, 8057 Zurich, Switzerland; 2 Kalahari Meerkat Project, Kuruman River Reserve, Northern Cape, South Africa

**Keywords:** close calls, cohesion, coordination, group split, meerkat, vocal hot spot

## Abstract

Group coordination, when ‘on the move’ or when visibility is low, is a challenge faced by many social living animals. While some animals manage to maintain cohesion solely through visual contact, the mechanism of group cohesion through other modes of communication, a necessity when visual contact is reduced, is not yet understood. Meerkats (*Suricata suricatta*), a small, social carnivore, forage as a cohesive group while moving continuously. While foraging, they frequently emit ‘close calls’, soft close-range contact calls. Variations in their call rates based on their local environment, coupled with individual movement, produce a dynamic acoustic landscape with a moving ‘vocal hotspot’ of the highest calling activity. We investigated whether meerkats follow such a vocal hotspot by playing back close calls of multiple individuals to foraging meerkats from the front and back edge of the group simultaneously. These two artificially induced vocal hotspots caused the group to spatially elongate and split into two subgroups. We conclude that meerkats use the emergent dynamic call pattern of the group to adjust their movement direction and maintain cohesion. Our study describes a highly flexible mechanism for the maintenance of group cohesion through vocal communication, for mobile species in habitats with low visibility and where movement decisions need to be adjusted continuously to changing environmental conditions.

## 1. Introduction

Members of cohesively foraging groups must stay within close range of their group mates [1,2] to achieve the benefits of group living. The mechanisms to reach group cohesion vary between species and are usually based on simple interaction rules such as attraction, repulsion and alignment between individuals, often achieved through visual cues, such as described for fish schools [3,4]. However, different mechanisms are needed in moving animal groups in structured habitats, when the location of an individual is highly dynamic and visual contact is limited. In these cases, other modalities of communication between individual group members become important to maintain group cohesion. Many bird and mammal species use vocalizations to reduce the risk of separation, especially during foraging [5,6]. ‘Close calls’, soft close-range contact calls, are frequently produced during group movement, and are thought to function primarily to maintain group cohesion [5–8]. When visibility is low, individuals often change the structure of their calls [9] or their call rate with increasing separation risk [5,7]. Most research has focused on changes in the vocal behaviour of individuals based on their relative spatial location to other group members [5–7,9], but has not addressed the specific mechanisms by which contact calls lead to group cohesion. Here, we investigated whether foraging meerkats (*Suricata suricatta*) use the distribution of close calls in the group (given by the number of individuals and their call rate at a specific location) and follow in the direction of ‘vocal hotspots’, areas with many closely aggregated individuals calling at high rates [10], to maintain cohesion during movement.

**Figure 1.**
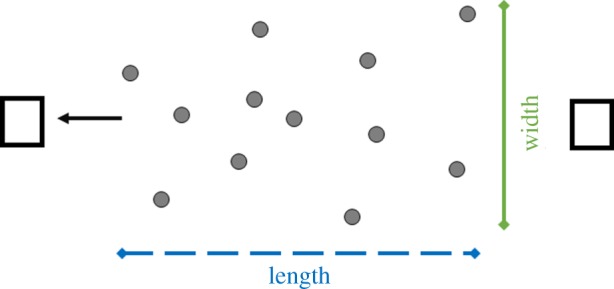
Diagram of the experimental set-up: the length (blue dashed line) and width (green line) of a group (grey points) in the direction of movement (black arrow) as estimated in our data. The black squares indicate the location of the speakers in relation to the group during each playback.

Meerkats, cooperatively breeding mammals, live in social groups of up to 50 individuals [11] which forage cohesively, typically 1–10 m next to each other [10]. Individuals search for prey in the sand with their heads orientated downwards [11], thus impairing visual communication. They instead rely on an array of vocal signals to coordinate their activities [12]. During foraging, close calls are the most frequently emitted vocalization and are thought to maintain group cohesion [10,12]. Meerkats adjust their call rates depending on their social environment [10,13,14] with call rates decreasing with increasing distance to their closest neighbour, and increasing towards the front of the group, relative to the direction of movement [10]. Thus, a spatial pattern of calls, areas with few individuals calling at low rates and areas with many individuals calling at high rates (vocal hotspot), emerges with a vocal hotspot typically towards the centre-front of the group [10]. Here, we hypothesize that meerkats use the vocal hotspot to determine where most group members are located relative to their own spatial location, thus guiding each individual's future movement towards them. To test this hypothesis, we manipulated the distribution of close calls in the group. We played back calls of multiple individuals from two sides, the front and the back edge of the group (figure 1), creating two artificial vocal hotspots, and moved them into opposite directions. If meerkats are guided by the distribution of close calls in the group in their future movement direction, we would expect individuals to follow the closest artificially induced vocal hotspot. Thus, we predict the group to elongate and/or split into two subgroups, as individuals at the front and back end of the group perceive two different vocal hot spots.

## 2. Material and methods

The study was conducted at the Kalahari Meerkat Project, Kuruman River Reserve, in the Northern Cape of South Africa. Data were collected from January to March 2015 on eight groups, ranging from 9 to 22 individuals (mean ± s.d. = 16 ± 4) during the morning foraging session. During the period of data collection, daily temperatures ranged from 10°C to 42°C and daily rainfall from 0.0 to 30.0 mm m^−2^ (with a median daily rainfall of 3.1 mm m^−2^ on a total of 16 days during the three months of data collection). A more detailed description of the climate and habitat are provided by Clutton-Brock *et al.* [15]. In contrast to winter when meerkats mostly forage continuously for the whole day, in summer meerkats forage for a couple of hours, starting just after dawn and in the evening before sunset, and rest during the hottest part of the day. All animals were visually identifiable through dye mark combinations [16] and were habituated to close human observation within less than 1 m.

### 2.1. Recordings and playbacks of close calls

To test whether meerkats follow areas with a high number of close calls, we performed a playback experiment, inducing two artificial vocal hotspots, areas with the highest calling rate. Calls, used in the playback sound files, were recorded using a directional microphone (Sennheiser ME66 with K6 powering module) connected to a recorder (Marantz PMD660, sampling frequency 44.1 kHz, 16 bit) at a distance of 0.3–1.5 m from the individual. Playback sound files were created for each group in Cool Edit Pro 2.0 (Syntrillium Software Corporation) by selecting high-quality calls from the recordings of the same group, where the playbacks were performed.

We created two 20 min playback tracks for each group with close calls of four adult subordinate group members. The call rate of each individual on the track reflected its ‘natural’ call rate, observed during previous foraging sessions and was the same for both playback tracks. However, to avoid playing exactly the same sound files back simultaneously from both loud speakers, we randomized the order of calls of recorded individuals within each track, by assigning two random numbers to each call, one for the time within the playback and one for the call itself. We played these close call sound files as test condition to eight meerkat groups from the front and back edges of the group simultaneously. As a control, we played background noise, recorded in the group's home range when no meerkats were present, with the same set-up. We played both conditions to a group during the same morning session (starting 30 min after the onset of foraging to midday), to avoid confounding time effects due to possible changes in habitat and climate between sessions. The sound files were played from a Marantz recorder connected to a speaker (X-Mini Uno XAM14) attached to the leg of the observers at about 15 cm above ground. The order in which we played the close calls (test condition) and the background noise (control condition) was counterbalanced, with at least 15 min between conditions.

During the playback, observers with the speakers stood at the edge of the group, maintaining a constant distance of 3 m to the closest meerkat. Hence, when meerkats moved towards the speaker, the speaker was slowly moved away from the group to maintain a constant distance. Throughout the playback, the observers recorded the behaviour of the group on a video camera (Sony Handycam 3.3MPXL). Every 2 min (time steps), we estimated the spatial width of the group as the distance between the furthest two animals at an angle of 90° to the direction of group movement. We also estimated the length of a group as the distance between the first and the last meerkat in the direction of group movement (figure 1). We further recorded the occurrence of subgroups, i.e. subclusters of at least three animals, as well as the distance (gap size) between the edges of these subgroups. To account for variation in overall group dispersion, we normalized the gap size by subtracting the average distance between neighbouring meerkats.

### 2.2. Statistical analysis

Statistical tests were carried out using R (v. 3.3.1) [17]. In order to investigate whether groups more likely split when playing back close calls, compared with background noise, we fitted three linear mixed effects models (LMMs) [18] with the gap size, the length and the width of the group as responses, and the interaction between the playback condition and the time steps within each playback as explanatory variables. The time steps refer to the 2 min intervals at which we documented the length and width of the group and the gap size between subgroups. The intercept of the model represents the predicted values for the control playback of background noise at time step zero. The coefficients of the model (‘estimate’ in table 1) give the difference in the mean of the levels of a categorical explanatory variable and the slope for continuous variables. It also indicates the direction of the effect of a factor (positive or negative) on the response variable. Gap size was log transformed before fitting the model to ensure the normal distribution of the data. We included the identity of the group as a random factor and accounted for multiple testing using a Bonferroni correction.

**Table 1.** Results of the LMMs testing how the length, width and the formation of subgroups were affected by the playback of close calls and the playback of background noise. Significant effects are highlighted in italics. Owing to the Bonferroni correction for multiple testing, the *α* level was reduced from 0.05 to 0.017 to account for multiple testing. The time steps are the 2 min intervals within the playback at which the length, width and gap size were estimated. The intercept of the model represents the predicted values for the control playback of background noise at time step 0. The coefficients of the model (estimate) give the difference in the mean of the levels of a categorical explanatory variable and the slope for continuous variables. It also indicates the direction of the effect of a factor (positive or negative) on the response variable.

response variable	explanatory variables	estimate	confidence intervals [0.025, 0.975]	s.e	d.f.	*t*-value	*p*-value
length (m)	intercept	16.84	[12.60, 21.09]	2.17	13	7.77	<*0*.*001*
	playback ‘cc’	7.84	[4.43, 11.25]	1.74	162	4.51	<*0*.*001*
	time step	0.33	[0.12, 0.54]	0.11	162	3.09	*0*.*002*
	playback ‘cc’ : time step	−0.26	[−0.56, 0.03]	0.15	162	−1.76	0.080
gap size (m)	intercept	0.39	[−0.17, 0.94]	0.28	33	1.36	0.183
	playback ‘cc’	0.90	[0.27, 1.53]	0.32	162	2.78	*0*.*006*
	time step	0.00	[−0.04, 0.04]	0.02	162	−0.13	0.898
	playback ‘cc’ : time step	0.01	[−0.05, 0.06]	0.03	162	0.28	0.778
width (m)	intercept	23.58	[19.07, 28.10]	2.30	23	10.24	<*0*.*001*
	playback ‘cc’	−1.58	[−6.27, 3.12]	2.40	162	−0.66	0.511
	time step	−0.05	[−0.33, 0.24]	0.15	162	−0.31	0.755
	playback ‘cc’ : time step	0.15	[−0.26, 0.55]	0.21	162	0.71	0.479

## 3. Results

**Figure 2.**
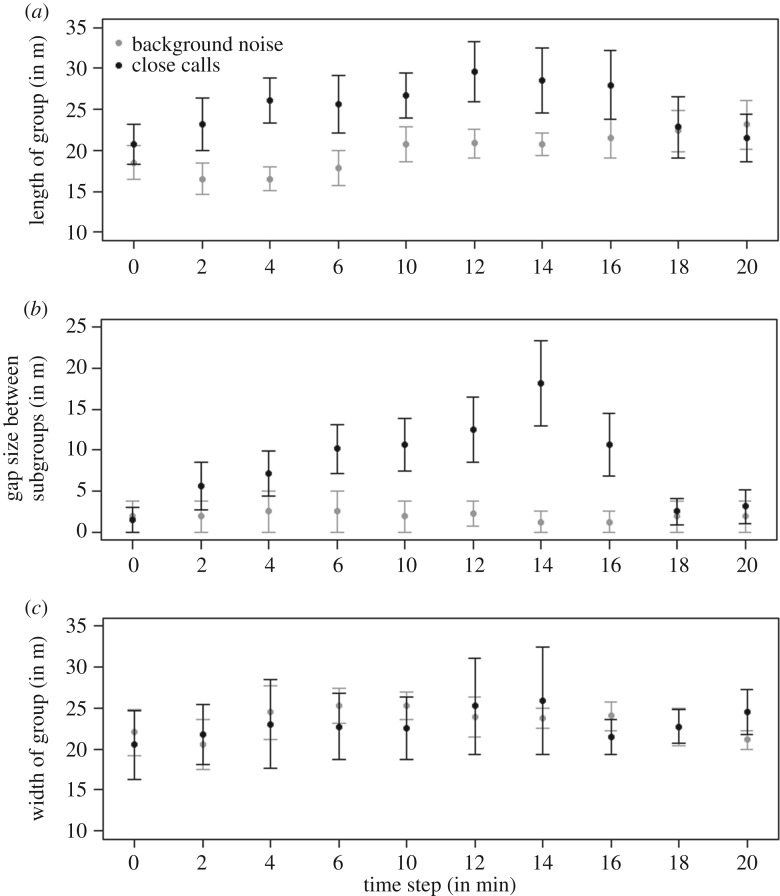
The mean (points) and standard deviation (whiskers) of a group's length (*a*), the gap size between subgroups (*b*) and its width (*c*) during the playback of background noise (grey) and the playback of close calls (black), measured at 2 min intervals throughout the experiment.

When artificially inducing two vocal hot spots by playbacks of close calls at the front and back edge in meerkat groups, individuals followed the nearby speaker, i.e. the closest location with the highest calling rate. Thus, the groups spatially elongated in the direction of the speakers, and the gap between subgroups became significantly larger when playing back close calls compared with background noise from the two opposite sides of the group (table 1 and figure 2*a*,*b*). However, subgroups reunited towards the end of the playback (figure 2*a,b*). Groups did not widen based on playback condition (table 1 and figure 2*c*). The time steps within each playback caused an elongation of groups, but did not influence the gap size, nor the width of the group. We found no interaction between the time step within each playback and the playback condition (table 1 and figure 2).

## 4. Discussion

Our experiment reveals that meerkats use close calls, constantly emitted by their group members while foraging, to make decisions about their own movement direction. Creating two artificial vocal hotspots of highest call rates by playing back close calls of several individuals at opposite sides of the group resulted in an increase in group elongation and a higher probability of splitting into two subgroups. This effect decreased after some time into the playback, likely due to individual meerkats of most groups realizing that they were getting separated and starting to produce alert calls, causing the two subgroups to reunite (G.E.C.G. 2015, personal observation).

Two different mechanisms might lead to groups splitting: individuals might orientate and follow towards the closest speaker, as the closest vocal hotspot, or individuals at the back of the group might move more slowly due to hearing many calls around them, but follow a general movement direction given by individuals in the front or being determined as commonly used foraging route. Given that not only the observer at the front of the group, but both observers slowly moved apart from each other and away from the closest meerkats, the first option is more likely. Meerkats followed the speakers carried by the observers, who decided on the movement direction (G.E.C.G. 2015, personal observation). Whether the movement direction of animals at the front of the group might have coincided with the direction chosen by the group is impossible to tell. However, the individuals in the back turned around to follow the speaker in the opposite direction. Thus, if we had played the calls from both sides instead of the front and back of a group, we would expect the same results. Following a moving vocal hot spot allows each individual to constantly direct towards the part of the moving group where likely several members aggregate, and ensures group cohesion even if visual contact is restricted due to vegetation and topographical barriers, and no general foraging direction or route governs a group's movement.

Group coordination has mainly been studied in the context of movement initiation; however, how social animals coordinate movement while moving with restricted visual contact has been more challenging to investigate. Meerkats adjust their close call rate based on their own relative spatial location within the group as well as their social environment, calling less with increasing distance to the closest neighbour, and at higher rates towards the front of the group as well as when in close proximity to dominant individuals [10,13]. By following the emergent vocal hotspot, meerkats thus adjust their movement direction presumably to avoid losing contact with their group. Our results support the findings for wild sooty mangabeys (*Cerocebus atys*) [19] that an individual's movement is influenced by its social environment and the call rates of other group members. Sooty mangabeys change their movement speed and potentially their direction depending on the call rate of their own subgroup and call rates of other primate species frequently associating with them. Nevertheless, it is unclear how individual mangabeys adjust their own call rate to their surroundings and contribute to the movement of others through their own vocal behaviour.

Based on prior knowledge and the results of this experimental study, we can integrate our understanding on both the production [10] and the receiver side of a moving vocal hotspot, defined as the location of the highest call rate within a group, and suggest it as a highly flexible coordination mechanism enabling adjustment to fast-changing movement decisions. This mechanism shows similarities to cliff swallow ‘squeak calls’ used to attract conspecifics to moving insect swarms [20]. In a more general context, the locations with highest densities of vocal signals function similar to ant pheromone trails to recruit and direct other group members to a specific location, though in the case of ants with stationary destinations [21]. This suggests that signal hotspots, consisting of vocalizations or signals of other modalities, provide a robust and flexible way for individuals to track the core of the group and maintain cohesion during foraging. Previous studies show that close call rate increases towards the front of the groups progression [10], suggesting that individuals located at the front might actively increase their call rate. Furthermore, meerkats are able to distinguish between close calls of different individuals [14] and might therefore take the identity of the callers into account when following a signal hotspot. Whether these potentially more informed individuals can ‘lead’ the group [19,20] by affecting the location of the signal hotspot with an increase in their own call rate, thereby influencing the movement of others, is yet to be explored.

## Data accessibility

Data are available via Dryad (http://dx.doi.org/10.5061/dryad.qb562) [22].
